# Double Burden of Malnutrition and the Relationship Between Reported Intestinal Parasitosis and Anemia in School-Aged Children from a Peri-Urban Community of Limpio (Paraguay): A Cross-Sectional Study

**DOI:** 10.3390/nu18132192

**Published:** 2026-07-05

**Authors:** María Teresa Murillo-Llorente, Javier Pérez-Murillo, Miriam Martínez-Peris, Alma María Palau-Ferré, Ignacio Ventura, María Ester Legidos-García, Jorge Casaña-Mohedo, Marcelino Pérez-Bermejo

**Affiliations:** 1 SONEV Research Group, Faculty of Medicine and Health Sciences, Catholic University of Valencia San Vicente Mártir, C/Quevedo No. 2, 46001 Valencia, Spain; mt.murillo@ucv.es (M.T.M.-L.); javier.perezmu@ucv.es (J.P.-M.); miriam.martinez@ucv.es (M.M.-P.); am.palau@ucv.es (A.M.P.-F.); ester.legidos@ucv.es (M.E.L.-G.); jorge.casana@ucv.es (J.C.-M.); 2 Translational Research Center San Alberto Magno CITSAM, Catholic University of Valencia San Vicente Mártir, C/Quevedo No. 2, 46001 Valencia, Spain; ignacio.ventura@ucv.es

**Keywords:** double burden of malnutrition, childhood obesity, anemia, intestinal parasites, stunting, nutritional status, schoolchildren, Paraguay

## Abstract

**Background/Objectives**: The nutrition transition in low- and middle-income countries has produced a double burden of malnutrition (coexistence of excess weight, undernutrition, and micronutrient deficiencies), with scarce evidence in schoolchildren from vulnerable peri-urban areas of Paraguay. The objective was to characterize, in a multidimensional way, the nutritional status of children and adolescents from Limpio and to explore its associations with anemia and clinical, dietary, and environmental variables, in particular, reported intestinal parasitosis. **Methods**: Cross-sectional observational study in 90 participants aged 6 to 16 years recruited by convenience at six community settings. Anthropometry, body composition, capillary hemoglobin, dietary patterns, and environment were assessed. Weight status was classified using the WHO 2007 references (z-scores), anemia was described using WHO thresholds, and central obesity was assessed using a waist-to-height ratio > 0.5. Non-parametric tests, Fisher’s exact test, Spearman correlations, and multivariable logistic regression were used. **Results**: Overweight or obesity affected 39.3% (obesity, 16.7%) and central obesity 22.4%, with no cases of thinness, coexisting with anemia (27.0%), stunting (8.2%), and reported intestinal parasitosis (24.1%). Anemia was more frequent in children with reported intestinal parasitosis (45% versus 20%; adjusted OR 5.44; 95% CI 1.44–20.51). Height-for-age was inversely associated with the number of siblings (ρ = −0.25). **Conclusions**: This population showed a double burden of malnutrition. The association between reported, non-laboratory-confirmed intestinal parasitosis and capillary-hemoglobin-defined anemia was exploratory and non-causal, given the cross-sectional design. Together with the high burden of anemia, these findings raise the hypothesis of a possible triple burden of malnutrition, which would require confirmation through stool parasitological testing and biomarkers of iron status, inflammation, and other micronutrients. These findings are compatible with integrated community strategies addressing dietary quality, sanitation, and access to safe water; decisions on deworming and micronutrient supplementation should be guided by local parasitological surveillance and biomarker-based assessment rather than by these data alone. Because the study used a convenience sample from a single peri-urban community during one fieldwork period, the findings should not be generalized beyond similar vulnerable settings without further confirmation.

## 1. Introduction

Child and adolescent nutrition is undergoing a complex epidemiological and dietary transition, particularly evident in low- and middle-income countries. Within a single setting, there is an increasingly frequent coexistence of persistent undernutrition (stunting, micronutrient deficiencies, and anemia, which continue to affect the most socially vulnerable groups) and a sustained rise in overweight and obesity in childhood and adolescence [1,2]. This coexistence constitutes the so-called double burden of malnutrition, which the literature describes at three non-mutually exclusive levels: the population level, in communities where high prevalences of weight deficit and excess co-occur; the household level, with the cohabitation of members with undernutrition and with obesity; and the individual level, when the same person presents excess adiposity together with nutritional deficiencies, anemia, or other markers of vulnerability [1,2]. Far from constituting successive stages of a single process, both forms of malnutrition tend to overlap, driven by the nutrition transition: accelerated changes in food systems, greater availability of low-cost, energy-dense products, and declines in physical activity [1].

Latin America and the Caribbean exemplify this transition, marked by urbanization, social inequality, and unequal access to preventive health services [3,4]. A systematic review focused specifically on the individual level estimated that the double burden of malnutrition in children and adolescents may range between 1.0% and 35.4%, with the highest figures in low- and middle-income countries and particular relevance in adolescence [5]. In the Latin American region, a recent meta-analysis documented that its different typologies reach relevant prevalences and that overweight constitutes one of the most frequent components [4], in a context in which chronic undernutrition is declining slowly while weight excess continues to rise [3]. This picture makes it necessary to shift the focus from a view centered exclusively on weight deficit toward a broader understanding of dietary quality, body composition, and early markers of vulnerability.

From this perspective, the double burden should not be interpreted solely as the mere community-level co-occurrence of underweight children and children with obesity. Its relevance lies in the fact that a sufficient (or even excessive) energy intake can coexist with low dietary diversity, high consumption of free sugars and ultra-processed foods, and deficiencies of iron or other micronutrients, a phenomenon that has been described as hidden hunger or hidden malnutrition [2,6]. In pediatric populations with normal weight, overweight, or obesity, body weight may mask diets of low nutritional density and specific deficiencies, so that an assessment based exclusively on weight is insufficient to describe nutritional status [6].

Among the markers of nutritional vulnerability, anemia occupies a prominent place. According to the Global Burden of Disease Study 2021, anemia affected close to a quarter of the world’s population and ranked among the leading causes of years lived with disability, with the greatest burden concentrated in early childhood, women of reproductive age, and low- and middle-income countries [7]. Its etiology is multifactorial and includes deficiency of iron and other micronutrients (folate, vitamins A and B12), hemoglobinopathies, chronic inflammation, and infections [7]. Therefore, anemia is not unequivocally equivalent to dietary iron deficiency, and its interpretation requires considering the clinical and environmental context in which it occurs.

Among infectious causes, intestinal parasitic infections (soil-transmitted helminths and protozoa) remain endemic in peri-urban settlements with poor sanitation and limited access to safe water and disproportionately affect the school-aged population of low- and middle-income countries [8]. Several cross-sectional studies in schoolchildren have described associations between intestinal parasitosis and a higher frequency of anemia, as well as with indicators of nutritional compromise, such as stunting, through biologically plausible mechanisms, such as intestinal blood loss, nutrient malabsorption, and chronic inflammation [8,9,10]. Nevertheless, given the cross-sectional nature of most of these works, such findings are interpreted as associations and do not allow causal relationships or their directionality to be established [9,10]. In this framework, periodic deworming and improved sanitation are considered among preventive strategies aimed at the school-aged population of endemic areas.

The school-aged and adolescent population (approximately from 6 to 18 years) has historically received less attention in nutritional surveillance, traditionally centered on children under five years, despite being a decisive stage that encompasses the pubertal growth spurt and the consolidation of dietary and physical activity habits. Oral health is also part of this comprehensive approach: childhood dental caries is related to frequent exposure to free sugars and sugary products and can be considered an indirect marker of unfavorable food environments, especially when it co-occurs with low dietary diversity or limited access to dental prevention [11].

Paraguay constitutes a setting of interest for analyzing this transition in the child and youth population, given that it combines the persistence of social and dietary vulnerability with a progressive increase in weight excess, in line with the regional trend [4]. Limpio, located in the Central Department and close to Asunción, brings together characteristics typical of territories in transition: urban growth, the presence of vulnerable settlements, inequality in access to healthy foods, sanitation limitations, and irregular access to preventive strategies, such as deworming. Studying children and adolescents linked to community canteens makes it possible to approach a population exposed to conditions that may co-occur both with nutritional deficiencies and, simultaneously, with excess adiposity.

For all these reasons, isolated anthropometric assessment is insufficient to characterize nutritional status in vulnerable pediatric populations. A more informative approach must integrate the dietary pattern, body composition, clinical signs compatible with possible deficiencies, oral health, blood pressure, capillary glucose, hemoglobin, and hematocrit. Likewise, in children and adolescents, it is essential to use age- and sex-specific classification criteria, such as the World Health Organization references for the population aged 5 to 19 years or the International Obesity Task Force cut-offs, since the direct application of adult body mass index cut-offs may induce relevant classification errors [12,13].

The main objective of this study was to characterize the nutritional status of children and adolescents from the community of Limpio (Paraguay) through a multidimensional assessment that included dietary, anthropometric, body composition, clinical–nutritional, oral, and hematological indicators. Specifically, the dietary pattern, clinical signs compatible with possible nutritional deficiencies, oral health status, anthropometric and body composition variables, blood pressure, oxygen saturation, capillary glucose, hemoglobin, and hematocrit were described; and the associations between nutritional status and anemia, on the one hand, and a set of clinical, dietary, behavioral, and environmental variables, on the other, were explored, with particular attention to the relationship between intestinal parasitosis, deworming, and anemia. Given the cross-sectional nature of the design, the results are presented in terms of associations and not of causal relationships or of risk or protective factors. The working hypothesis was that this vulnerable pediatric population would present a mixed nutritional profile, compatible with low dietary quality and the double burden of malnutrition rather than a one-dimensional pattern defined exclusively by weight deficit or excess.

## 2. Materials and Methods

### 2.1. Design, Setting, and Participants

An observational, descriptive, cross-sectional, exploratory study was conducted, aimed at characterizing the nutritional status and the main clinical–nutritional markers of a community sample of children and adolescents from Limpio (Central Department, Paraguay). Data collection was carried out between 10 and 31 July 2024 in community educational settings set up for the assessment, within the framework of a health cooperation activity developed by the Faculty of Medicine and Health Sciences of the Universidad Católica de Valencia San Vicente Mártir (Spain). The study was reported in accordance with the recommendations of the STROBE Statement (Strengthening the Reporting of Observational Studies in Epidemiology) for cross-sectional observational studies; the complete STROBE checklist is provided in Supplementary Materials (Table S1).

The source population consisted of children and adolescents residing in Limpio or linked to the selected school premises and community educational settings. Recruitment was carried out through a non-probabilistic convenience community strategy, conditioned by community accessibility, children’s attendance at assessment points, and the availability of informed consent. Prior to the fieldwork, the research team coordinated the sessions with local liaisons, who facilitated communication with families and logistical organization.

Participants aged from 6 to 16 years, apparently healthy at the time of assessment, whose parents or legal guardians authorized their participation through informed consent, were included. Excluded were children not present during the sessions, those in whom it was not possible to complete the basic measurements, and those with a known chronic disease or pharmacological treatment that could modify nutritional status, body composition, or hematological parameters, when this information was available. Capillary determinations of glucose, hemoglobin, and hematocrit were performed only in participants with valid measurements and specific authorization.

Of a total of 238 children and adolescents identified as the accessible community population at the six recruitment points, 90 were finally assessed (coverage of 37.8%). The main reasons for non-inclusion were non-attendance during the sessions, the absence of family authorization, and the impossibility of completing the basic measurements. The community origin is detailed in Table 1.

### 2.2. Data Collection and Instruments

Data were collected using a standardized field booklet specifically designed for the community assessment of nutritional and clinical–nutritional status, which allowed sociodemographic, perinatal, environmental, dietary, lifestyle, anthropometric, clinical, and basic biochemical information to be recorded in a homogeneous manner. Data collection was carried out by a health team composed of a physician and community nurses, previously coordinated to standardize the procedures for interview, examination, anthropometric measurement, and recording of vital signs. The interviews were adapted to the participant’s age and, when necessary, were completed with the support of the mother, father, or legal guardian.

### 2.3. Sociodemographic, Perinatal, and Environmental Variables

Sociodemographic variables included age (in completed years), sex (according to the information provided by the participant or their guardian), academic grade, number of siblings, and the community recruitment point. Perinatal and early-childhood background, the duration of exclusive breastfeeding, gestational age at birth, birth weight, and Apgar score were recorded, based on the guardian’s recall and the available health documentation. Environmental conditions comprised the type of housing, source of water supply, and other household characteristics related to sanitation. Likewise, reported information on vaccination status, micronutrient supplementation, and performance of deworming was collected by interview. The history of intestinal parasitosis was obtained by asking the adult accompanying the child whether the health center had previously informed them of the presence of intestinal parasites; the research team did not perform a stool parasitological examination or review clinical documentation, so this variable reflects a history of diagnosis reported by the participant, usually established at the health center in the presence of symptoms, and not a systematic parasitological determination carried out within the framework of the study. Given their reported nature, all these variables are interpreted with the corresponding caution.

### 2.4. Dietary and Lifestyle Assessment

The dietary pattern was assessed using two 24 h recalls administered by structured interview during the field sessions. In younger participants, the information was provided by the mother, father, or legal guardian, or completed with their help; in adolescents, the interview was conducted directly with the participant and cross-checked, when possible, with the accompanying adult. Each recall collected the foods and beverages consumed the previous day, distinguishing breakfast, lunch, afternoon snack, dinner, and between-meal consumption. Quantities were estimated approximately using common household measures (glasses, cups, spoonfuls, units, plates, or portions reported by the participant) and, when necessary, with the support of simple visual examples, without systematic conversion to grams or quantitative nutritional analysis. Given the exploratory nature of the study, the recalls were not used to estimate individual habitual intake or the intake of energy, macronutrients, or micronutrients, but only to obtain a qualitative impression of the general consumption pattern.

For this descriptive purpose, the recorded foods and beverages were arranged into general categories (fruits, vegetables, cereals and tubers, protein foods, dairy products, sugary beverages, non-sugary beverages or water, sweet or pastry products, salty snacks, and ultra-processed preparations or those of low nutritional density), used to qualitatively characterize the dietary pattern of the sample. No quantitative indicators, such as consumption frequencies by group or dietary diversity indices, were derived from the recalls.

The quantitative indicator of dietary quality was a structured questionnaire of 12 dichotomous items adapted from the KIDMED index [14], summarized as the number of favorable responses (0–12). It must be emphasized that this is a non-validated adaptation of the original 16-item instrument and that, in a non-Mediterranean context, its cross-cultural validity is limited; therefore, this score should not be interpreted as a measure of actual adherence to the Mediterranean diet, but as a descriptive proxy of dietary quality based on the presence of healthy and unhealthy food items. Physical activity was recorded through reported participation (yes/no) and, when applicable, its type, intensity, frequency, and duration; daily water intake (liters) was also collected. Psychological well-being was assessed using a scale derived from the Ryff model, with six dimensions scored from 0 to 10 (total score from 0 to 60) [15].

### 2.5. Clinical–Nutritional Assessment and Vital Signs

The clinical–nutritional assessment included the systematic observation of skin, hair, nails, and oral cavity. Visible signs compatible with possible nutritional vulnerability or with a need for follow-up were recorded, such as dry or macerated skin, weak hair, brittle or fragile nails, lip fissures, oral mucosa alterations, gingivitis, dental stains, malocclusion, and lesions compatible with dental caries. These findings were considered indicators for community screening and surveillance, without isolated etiological diagnostic value; in particular, lesions compatible with caries were recorded by field visual inspection and do not replace a complete dental evaluation. The health team also recorded systolic (SBP) and diastolic (DBP) blood pressure, heart rate, arterial oxygen saturation (SaO_2_), capillary glucose, hemoglobin, and hematocrit. An Omron M3 blood pressure monitor with different cuff sizes (Omron Healthcare Co., Ltd., Kyoto, Japan), a Beurer PO 80 pulse oximeter (Beurer GmbH, Ulm, Germany), a Contour XT glucometer (Ascendia Diabetes Care, Basel, Switzerland), and a HemoControl analyzer (EKF Diagnostics, Cardiff, United Kingdom) for hemoglobin and hematocrit were used. Capillary determinations were performed only in participants with family authorization, sufficient cooperation, and adequate conditions, so the number of valid observations varied across variables; in the results, each analysis uses the corresponding denominator of valid cases, without imputations or extrapolations. Biomarkers of iron status (e.g., serum ferritin, transferrin saturation, soluble transferrin receptor), systemic inflammation (e.g., *C*-reactive protein, α1-acid glycoprotein), and other micronutrients (e.g., vitamin B12, folate, vitamin A) were not determined because of the logistic and operational constraints inherent to this brief community-based field cooperation activity in a resource-limited peri-urban setting, in which only point-of-care capillary determinations were feasible. The implications of this limitation for the interpretation of anemia etiology and of the triple burden hypothesis are discussed in Section 4.7.

### 2.6. Anthropometry and Body Composition

The anthropometric assessment followed standardized procedures. Weight was measured with the participant barefoot and in light clothing, using a calibrated scale (Omron BF511; Omron Healthcare Co., Ltd., Kyoto, Japan), and height with a portable stadiometer (Seca 213; seca GmbH & Co. KG, Hamburg, Germany), in a standing position, with the heels together and the head in the Frankfurt plane. The body mass index (BMI) was calculated as weight (kg) divided by height squared (m^2^). In addition, indicators of body composition and adipose distribution were recorded: percentages of lean mass and fat mass, waist, mid-upper-arm, hip, and calf circumferences, and the biceps, triceps, subscapular, suprailiac, and abdominal skinfolds. Circumferences were measured with a flexible, non-extensible measuring tape and skinfolds with a caliper (Slim Glide Skyndfold Caliper; Creative Health Products, Plymouth, MI, USA), on the non-dominant side of the body. The inclusion of circumferences and skinfolds complemented the information from BMI, given that these indicators capture different dimensions of adiposity and its distribution in the pediatric age [16,17]. The waist-to-height ratio was calculated from the waist circumference and height [18].

### 2.7. Classification of Nutritional Status and Definitions

The main classification of weight status was based on the World Health Organization (WHO) growth references for the population aged from 5 to 19 years, using the z-scores of BMI-for-age (BAZ) and height-for-age (HAZ) [12]. The z-scores were calculated using the LMS method from the official coefficients of the WHO 2007 reference, expressing age in months. The WHO cut-offs were applied: thinness (BAZ < −2 standard deviations [SD]), normal weight (−2 to +1 SD), overweight (>+1 to +2 SD), and obesity (>+2 SD); stunting was defined as HAZ < −2 SD. Complementarily, the pediatric cut-offs of the International Obesity Task Force (IOTF) were considered, which classify thinness, overweight, and obesity from BMI trajectories anchored to the adult thresholds at 18 years [13]; the adult BMI criteria were not used for nutritional classification. Central obesity was defined as a waist-to-height ratio greater than 0.5 [18].

Anemia was defined using the WHO age- and sex-specific hemoglobin thresholds (<11.5 g/dL from 5 to 11 years; <12.0 g/dL from 12 to 14 years; and, from 15 years onward, <12.0 g/dL in females and <13.0 g/dL in males) [7]. Low birth weight was defined as a weight below 2500 g and prematurity as a gestational age below 37 weeks.

### 2.8. Statistical Analysis

Data processing and cleaning were performed in a spreadsheet (Microsoft Excel) and the statistical analyses in Python (version 3.12; pandas, SciPy, and statsmodels libraries). Quantitative variables were described using mean and standard deviation when their distribution was approximately symmetric and using median and interquartile range (IQR) in the case of asymmetry; qualitative variables were described using absolute frequencies and percentages, using the number of valid cases as the denominator. For the main clinical and nutritional proportions, 95% confidence intervals were calculated when the number of observations allowed it.

The normality of the quantitative variables was assessed by graphical inspection and the Shapiro–Wilk test. Comparisons by sex were performed with Student’s *t*-test for independent samples when the assumptions of normality and homogeneity of variances were met and, otherwise, with the Mann–Whitney U test. Associations between qualitative variables were examined with Fisher’s exact test, and associations between quantitative variables with the Pearson or Spearman correlation coefficients, depending on the distribution. To examine the association between anemia and intestinal parasitosis, a multivariable logistic regression model was fitted that included age and sex as covariates, presenting odds ratios with their 95% confidence intervals. As a sensitivity analysis of the anemia model, in addition to the model adjusted for age and sex, the unadjusted odds ratios (bivariate models) and a penalized logistic regression model according to the Firth method were estimated, suitable in the presence of a limited number of events per variable and the possible presence of cells with low frequencies.

The significance level was set at *p* < 0.05 (two-tailed). Given the cross-sectional design, the moderate sample size, and the exploratory nature of the study, the inferential analyses are interpreted as hypothesis-generating and not as confirmatory evidence; consequently, the findings are expressed in terms of associations and not of causal relationships or of risk or protective factors, and no direct inferences are made about the child and adolescent population of Limpio as a whole. As an additional sensitivity analysis for potential unmeasured confounding, E-values were computed for both the point estimate and the lower bound of the 95% confidence interval of the adjusted odds ratio, following the method of Ding and VanderWeele [19]. The E-value represents the minimum strength of association on the odds-ratio scale that an unmeasured confounder would need to have with both the exposure and the outcome, above and beyond the measured covariates, to fully explain the observed association. This analysis was not intended to correct for exposure misclassification, which remains possible because intestinal parasitosis was not confirmed by stool examination.

### 2.9. Ethical Considerations

The study was approved by the Research Ethics Committee of the Universidad Católica de Valencia San Vicente Mártir (code UCV/2023-2024/102; approved on 28 May 2024) and was conducted in accordance with the principles of the Declaration of Helsinki and with the applicable regulations on research involving human beings, personal data protection, and confidentiality. Written informed consent was obtained from the parents or legal guardians of all participants and, when applicable, the assent of the minors. Participation was voluntary, and participants could withdraw at any time without any consequence. The data were de-identified before analysis and treated confidentially, without including identifying information in the analytical database. The measurements performed were non-invasive or minimally invasive, limited to procedures of nutritional, anthropometric, and basic clinical assessment, and capillary determinations when family authorization and participant cooperation were available.

## 3. Results

### 3.1. Sociodemographic Characteristics of the Sample

Ninety school-aged children were assessed (median age 9.5 years; IQR 8.0–11.0; range 6–16), of whom 56 (62.2%) were girls. Half of the sample (50.0%) was between 6 and 9 years of age. The participants came from six peri-urban communities of the Limpio district linked to community canteens, which reflects a context of socioeconomic vulnerability. Most lived in brick housing (82.2%) and had piped water (75.6%), although a relevant proportion resorted to a well (12.2%) or other sources. Exclusive breastfeeding reached the recommended six months in 55.6% of cases, and only 2.2% received micronutrient supplementation. Self-reported intestinal parasitosis affected 24.1%, and almost a third (30.7%) had not been dewormed. The sociodemographic and environmental characteristics are detailed in Table 2.

### 3.2. Anthropometric, Clinical, and Lifestyle Descriptives by Sex

The anthropometric and hematological parameters, vital signs, and lifestyle indicators are summarized in Table 3, overall and stratified by sex. No statistically significant differences were observed between boys and girls in any of the analyzed variables (all *p*-values > 0.05), including the z-scores of BMI-for-age (BAZ) and height-for-age (HAZ), hemoglobin, and the indicators of central adiposity. Glucose showed a tendency to be higher in boys (*p* = 0.052), without reaching significance.

### 3.3. Nutritional Status: Predominance of Excess Weight

**Figure 1:**
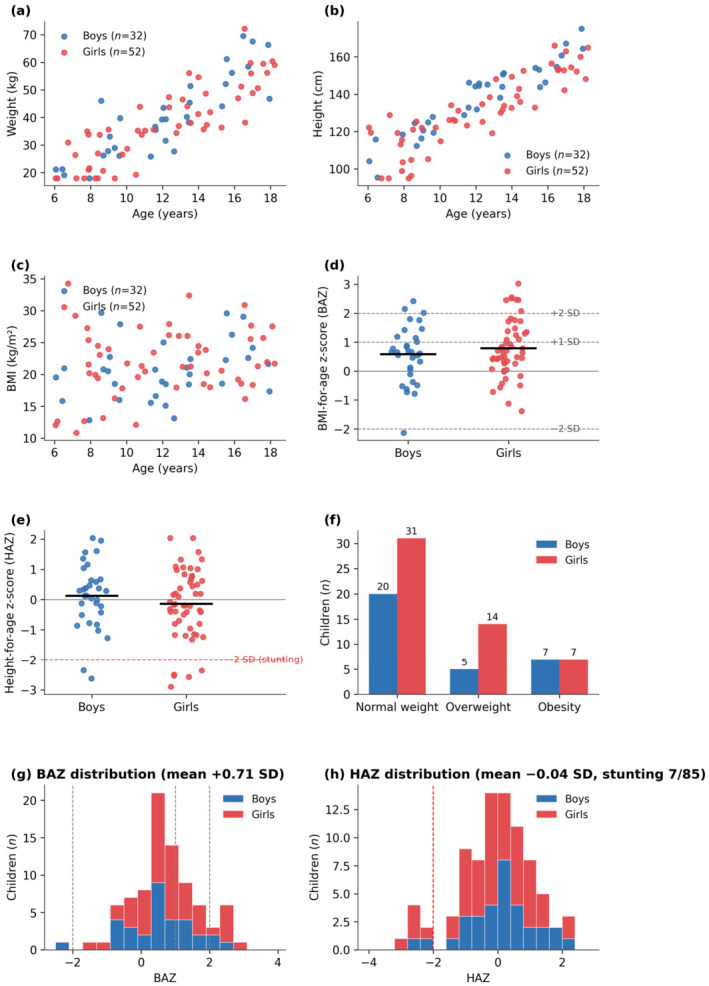
Anthropometric and growth indicators of the sample (*n* = 90), displayed with individual participant data points stratified by sex: (**a**) weight, (**b**) height, and (**c**) body mass index by age in years. (**d**) BMI-for-age z-scores (BAZ) shown as individual values per sex with the median (black bar) and the WHO reference lines at −2, +1, and +2 SD; (**e**) height-for-age z-scores (HAZ) by sex with the −2 SD stunting threshold; (**f**) absolute counts of children in each weight-status category by sex; (**g**) stacked BAZ distribution by sex (sample mean +0.71 SD); (**h**) stacked HAZ distribution by sex (sample mean −0.04 SD; 7/85 children below the stunting threshold). Boys are shown in blue and girls in red.

Excess weight dominated the distribution of BMI-for-age (Figure 1). Of the 84 children with a valid BAZ, 51 (60.7%) had normal weight, 19 (22.6%) were overweight, and 14 (16.7%) were obese; no child met the criterion for thinness. The mean BAZ was shifted upward (+0.71 SD), confirming a distribution of adiposity skewed to the right. The distribution of categories was similar in both sexes (*p* > 0.05). In parallel, height-for-age was situated close to the reference median (mean HAZ −0.04 SD), although the lower tail extended below −3 SD and 7 children (8.2%) presented stunting.

### 3.4. Coexisting Forms of Malnutrition (Double Burden)

**Figure 2:**
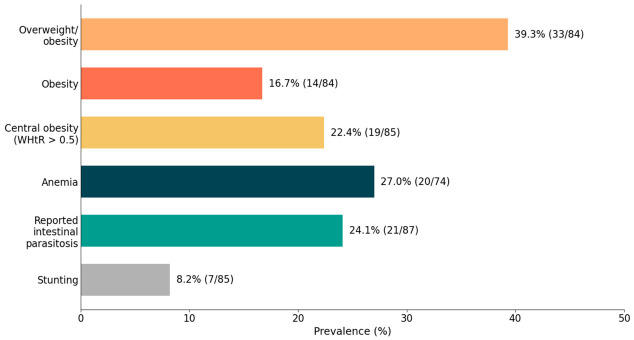
Prevalence of the coexisting forms of malnutrition, illustrating the double burden: excess weight and central obesity together with anemia, reported intestinal parasitosis, and stunting.

The population exhibited a clear double burden of malnutrition (Figure 2; Table 4). Excess weight (39.3%) and central obesity (22.4%) coexisted with stunting (8.2%) and a high burden of anemia (27.0%), against a background of frequently reported intestinal parasitosis (24.1%). Anemia coexisted with excess weight in 10 children: 36% of the children with overweight or obesity had anemia, compared with 21% of those with normal weight, which illustrates the double burden at the individual level, although this difference did not reach significance (*p* = 0.27).

### 3.5. Reported Intestinal Parasitosis and Its Association with Anemia

**Figure 3:**
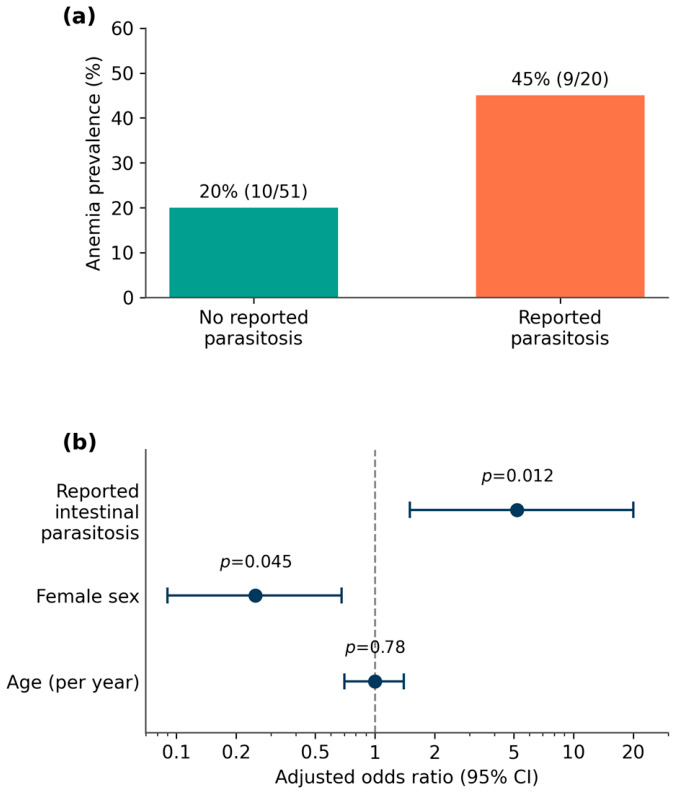
(**a**) Prevalence of anemia according to reported intestinal parasitosis status; (**b**) adjusted odds ratios (95% CI) from the multivariable logistic regression model for anemia.

The association of greatest magnitude was observed between reported intestinal parasitosis and anemia (Figure 3). It must be emphasized at the outset that intestinal parasitosis was ascertained by parental report of a previous diagnosis at the local health center and that the study team did not perform stool parasitological examination, so the following estimates may be affected by misclassification; the implications of this for the magnitude and direction of the association are discussed in Section 4.3 and Section 4.7. Anemia affected 45% (9/20) of the children with parasitosis versus 20% (10/51) of those without parasitosis (Fisher’s exact test OR 3.35; *p* = 0.040). The logistic regression model was based on 71 participants with complete data and 19 anemia events (6.3 events per variable). After adjustment for age and sex, reported intestinal parasitosis remained associated with anemia (adjusted OR 5.44; 95% CI 1.44–20.51; *p* = 0.012), although the magnitude of this estimate should be interpreted cautiously because of the limited number of anemia events and the wide confidence interval. A Firth-penalized logistic regression performed as a sensitivity analysis because of the limited number of events yielded a similar estimate (OR 4.82; 95% CI 1.33–17.49; *p* = 0.017). Female sex was associated with lower odds of anemia in the adjusted model (OR 0.26; 95% CI 0.07–0.97; *p* = 0.045), although this association did not hold in the penalized model (OR 0.29; 95% CI 0.08–1.04; *p* = 0.058) and should be interpreted with caution; age showed no association (Table 5). A descriptive, non-significant pattern was also observed for deworming: of the 21 children with reported intestinal parasitosis, 17 (81%) had not been dewormed, and anemia was more frequent in non-dewormed than in dewormed children (42% versus 21%; *p* = 0.093). Given the cross-sectional design and the lack of laboratory confirmation, this pattern should be interpreted cautiously and cannot establish directionality or effectiveness of deworming. The robustness of the parasitosis–anemia association to potential unmeasured confounding was explored using E-values [19]. For the adjusted odds ratio (5.44), the E-value was 10.35, indicating that an unmeasured confounder would need to be associated with both reported intestinal parasitosis and anemia by odds ratios of at least 10.35 each, above and beyond the measured covariates, to fully explain away the point estimate. For the lower bound of the 95% confidence interval (1.44), the corresponding E-value was 2.24, indicating the strength of confounding required to move the interval estimate to include the null. The Firth-penalized estimate (OR 4.82) yielded an E-value of 9.11 for the point estimate. These results provide a quantitative assessment of sensitivity to unmeasured confounding, but they do not correct for possible exposure misclassification. Therefore, the reported nature of the parasitosis variable remains an important limitation, and the association should be interpreted as exploratory.

### 3.6. Linear Growth, Number of Siblings, and Correlations

**Figure 4:**
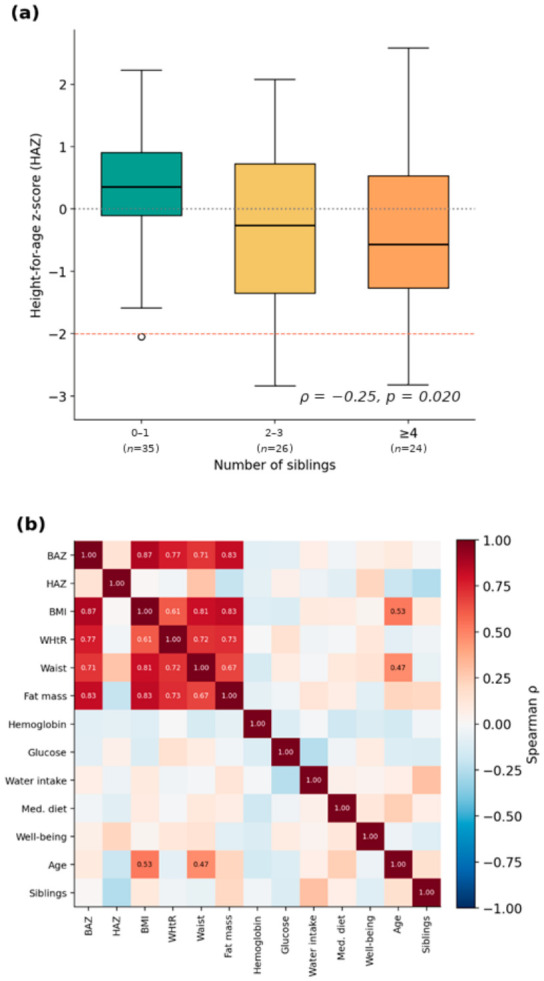
(**a**) Height-for-age z-score according to the number of siblings (box plots; the stunting threshold of −2 SD is shown). (**b**) Spearman correlation matrix of anthropometric, hematological, dietary, and behavioral variables.

Height-for-age decreased as the number of siblings increased (Spearman ρ = −0.25; *p* = 0.020): mean HAZ declined from +0.36 SD in children with 0–1 siblings to −0.30 SD in those with 2–3 siblings and −0.35 SD in those with ≥4 siblings, with a between-group difference at the limit of significance (Kruskal–Wallis *p* = 0.057), consistent with a possible gradient of resource dilution (Figure 4a). As expected, the adiposity indicators showed strong intercorrelation: BAZ was closely correlated with fat mass (ρ = 0.82; *p* < 0.001), the waist-to-height ratio (ρ = 0.77; *p* < 0.001), and waist circumference (ρ = 0.71; *p* < 0.001), which supports the internal consistency of the anthropometric data (Figure 4b). Dietary quality, according to the non-validated adapted KIDMED proxy, was low (median of 2 favorable items out of 12; 35.6% reported none), but neither the diet score (ρ = −0.03; *p* = 0.808), water intake, nor psychological well-being correlated significantly with BAZ. No association was found between the duration of breastfeeding and HAZ either (ρ = 0.09; *p* = 0.400).

## 4. Discussion

This cross-sectional exploratory study multidimensionally characterized the nutritional status of 90 children and adolescents from a vulnerable peri-urban community of Limpio (Paraguay) by integrating anthropometric, body composition, clinical–nutritional, hematological, dietary, and environmental indicators. The set of findings is consistent with a double burden of malnutrition at the community level: a clear predominance of excess weight (overweight or obesity in 39.3%) and central adiposity (22.4%) coexisted with the persistence of deficits (stunting in 8.2% and anemia in 27.0%) and with a notable absence of thinness. Far from describing a one-dimensional pattern, these results place the study population at the confluence of the different forms of malnutrition that characterize the nutrition transition in Latin America and the Caribbean [1,4].

### 4.1. Predominance of Excess Weight and Central Adiposity

The shift in the BMI-for-age distribution toward values above the reference median (mean z-score of +0.71 SD) and the high frequency of central adiposity, defined by a waist-to-height ratio greater than 0.5, indicate that weight excess constitutes the dominant component of the nutritional profile of this sample. This pattern is compatible with the nutrition transition described in the region, in which the greater availability of energy-dense, nutrient-poor foods and changes in lifestyles are accompanied by an increase in childhood weight excess [1,4]. The low dietary quality observed according to the adapted KIDMED proxy (with a median of 2 favorable items out of 12 and more than a third of participants without any favorable item) provides a dietary substrate consistent with this phenomenon, although the cross-sectional nature and the adapted nature of the instrument do not allow dependency relationships to be established [14].

The close correlation observed between the BMI-for-age z-score and the waist-to-height ratio (ρ = 0.77), as well as with fat mass (ρ = 0.82) and waist circumference (ρ = 0.71), supports the internal consistency of the anthropometric assessment and the usefulness of the waist-to-height ratio as a simple, non-invasive, and low-cost screening tool for central adiposity in resource-limited settings [18]. Adiposity in childhood and adolescence tends to be associated with early cardiometabolic markers and to persist throughout life; nevertheless, the design of the present study does not allow clinical outcomes or trajectories to be assessed, so these considerations should be understood as contextualization and not as inferences derived from our data [2].

### 4.2. Persistence of Growth Deficits and Gradient by Sibship Size

The simultaneous presence of 8.2% stunting, with a lower tail of the height-for-age distribution that extended below −3 SD, reflects the persistence of chronic undernutrition in a setting where excess weight already predominates. This coexistence is precisely the expression of the double burden and is consistent with the unfinished agenda of child undernutrition described for low- and middle-income countries [2,3]. The gradient observed between height-for-age and the number of siblings (with a decline in mean z-score from +0.36 SD in children with 0–1 siblings to −0.35 SD in those with four or more) is compatible with the resource dilution hypothesis, according to which the nutritional and economic resources of the household are distributed among a larger number of cohabitants. This relationship, modest in magnitude and at the limit of significance between groups, is interpreted as an association of a socioeconomic nature and not as a causal relationship.

### 4.3. Anemia and Reported Intestinal Parasitosis: The Most Marked Association

The most marked finding of this study was the association between reported intestinal parasitosis and anemia. Anemia affected 45% of children with parasitosis versus 20% of those without parasitosis, and the association remained after adjustment for age and sex (adjusted odds ratio of 5.44; 95% CI 1.44–20.51) and was robust in Firth-penalized regression analyses. This result is consistent with what has been described in schoolchildren from settings with poor sanitation, both in Latin America and in other low- and middle-income regions, where intestinal parasitosis has been related to a higher frequency of anemia and indicators of nutritional compromise [8,9,10]. The biologically plausible mechanisms include intestinal blood loss associated with hematophagous helminths, nutrient malabsorption, and low-grade chronic inflammation, which together may compromise the availability of iron for erythropoiesis.

The association extended to preventive care: of the 21 children with parasitosis, 17 (81%) had not been dewormed, and anemia was more frequent in the non-dewormed than in the dewormed (42% versus 21%). Taken together, these data outline a coherent pattern involving the absence of deworming, parasitosis, and anemia; nevertheless, the cross-sectional design precludes establishing the directionality of these relationships, which must be interpreted as associations and not as a demonstrated causal sequence. This caution is especially pertinent in light of several nuances in our own data: the mean hemoglobin concentration did not differ significantly between groups (medians of 12.0 versus 12.6 g/dL), so the association is based on the proportion of children crossing the anemia threshold rather than on a shift in the mean; and parasitosis and deworming were collected on a reported basis, without stool parasitological confirmation, which may introduce misclassification. The lower probability of anemia observed in girls should be interpreted with caution, given the small number of cases. It should also be added that evidence from systematic reviews indicates that community deworming administered in isolation has little or no effect on hemoglobin at the population level [20], which reinforces both the prudence in causal interpretation and the appropriateness of framing deworming within integrated strategies.

### 4.4. Anemia and the Hypothesis of a Possible Triple Burden of Malnutrition

The high prevalence of anemia, as well as excess weight and growth deficits, may suggest an additional dimension of nutritional vulnerability; however, because only capillary hemoglobin was measured, this should be interpreted cautiously, as a hypothesis related to a possible triple burden of malnutrition rather than as a demonstrated finding. This interpretation, however, must be framed explicitly as a hypothesis and not as a conclusion: in the present study, only hemoglobin was determined, and anemia is not unequivocally equivalent to a confirmed iron deficiency. In fact, its association with parasitosis suggests a probable mixed etiology, in which deficiency-related and infectious–inflammatory components would coexist.

In this framework, it is especially illustrative that anemia coexisted with excess weight in 10 children, and that its frequency was even somewhat higher among children with overweight or obesity (36%) than among those with normal weight (21%), although without reaching statistical significance. This apparently paradoxical coexistence (the double burden expressed in the same individual) is biologically plausible: in obesity, low-grade chronic inflammation induces the synthesis of hepcidin, which retains iron in macrophages and reduces its availability for erythropoiesis, favoring a functional iron deficiency [21]. The very low proportion of children receiving micronutrient supplementation (2.2%) is consistent with an environment of low nutritional density [6]. Confirmation of this triple burden hypothesis, however, would require the determination of biomarkers of iron status and of other micronutrients, which the present study lacks. We therefore wish to make this caveat explicit: the triple burden interpretation should be read as a hypothesis generated by the convergence of three indirect signals (excess adiposity, growth deficits, and a high prevalence of capillary-hemoglobin-defined anemia), and not as a finding of this study. The absence of biomarkers that would differentiate iron-deficiency anemia from anemia of inflammation of parasitic origin or of other etiologies (ferritin, transferrin saturation, soluble transferrin receptor, *C*-reactive protein, vitamin B12, folate) means that we cannot establish which specific micronutrient deficiencies underlie the anemia observed in this population. Future studies in this setting should incorporate these biomarkers, ideally with an adjustment for inflammation, in order to test the triple burden hypothesis directly rather than inferring it from anemia prevalence alone.

### 4.5. The Double Burden at the Individual Level and the Limits of Weight-Based Assessment

Taken together, the findings illustrate how body weight may mask diets of low nutritional density and specific deficits, so that an assessment based exclusively on weight or on the body mass index would be insufficient to describe the nutritional status of this population. The integration of anthropometry with body composition, clinical signs, hemoglobin, and environmental variables made it possible to simultaneously capture excess adiposity and markers of nutritional vulnerability, in line with the contemporary conception of malnutrition as a multidimensional phenomenon that encompasses both deficit and excess [2,5,6].

### 4.6. Public Health Implications

The observed mixed profile supports the appropriateness of integrated interventions, as opposed to programs centered on a single form of malnutrition. The framework of the so-called “double-duty actions,” which seek to simultaneously address undernutrition and excess weight by taking advantage of their common determinants, offers especially pertinent guidance for contexts such as the one studied [22]. In practice, plausible strategies would combine the promotion of higher-quality diet and physical activity with the improvement of sanitation and access to safe water, while deworming and micronutrient supplementation should be considered only where supported by local parasitological surveillance and biomarker-based assessment. Periodic deworming and micronutrient supplementation should not be inferred as recommendations from the present cross-sectional data alone, since (i) intestinal parasitosis was based on parental report rather than on parasitological confirmation, and (ii) the etiology of anemia could not be characterized in the absence of iron-status and inflammation biomarkers. Decisions on these specific measures should be informed by local parasitological surveillance, biomarker-based micronutrient assessment, and the existing evidence base on their effectiveness. The finding that deworming administered in isolation has limited effects on hemoglobin at the population level [20] underscores that such measures would yield more within integrated packages of water, sanitation, and hygiene combined with nutritional interventions than as independent actions. Schools and community canteens, through which the population was reached, constitute ideal platforms for deploying these strategies. Finally, the frequent recording of lesions compatible with dental caries, as an indirect marker of unfavorable food environments and of exposure to free sugars, reinforces the relevance of integrating oral health into community nutritional interventions [11].

### 4.7. Strengths and Limitations

Among the strengths of the study, the following stand out: its multidimensional nature, which combined standardized anthropometry, body composition, clinical examination, objective determination of hemoglobin, and dietary and environmental variables in a population that is difficult to access; the use of the official WHO 2007 references and the calculation of z-scores using the LMS method from their coefficients; and a transparent handling of the data, with recalculation of the body mass index, recoding of null physiological values as missing data, and exclusion of biologically implausible values without resorting to imputation.

Nevertheless, several limitations must be acknowledged. First, the cross-sectional design precludes establishing causal relationships or their directionality, so all findings are expressed as associations. Second, the sampling was non-probabilistic and by convenience, with a moderate sample size (*n* = 90), which limits statistical power, widens the confidence intervals (particularly in the logistic regression model), and precludes representative inferences for the child and adolescent population of Limpio as a whole. Third, key variables such as intestinal parasitosis, deworming, dietary pattern, and perinatal background were collected on a reported basis, without laboratory or systematic documentary confirmation by the team, which may introduce classification, recall, and detection or care-seeking biases. In the case of parasitosis, the datum corresponds to a history of diagnosis reported by the participant, usually established at the health center in the presence of symptoms. Therefore, the exposure may over-represent children whose families sought care or had better access to health services, while children without a reported diagnosis could still harbor undetected infections, particularly asymptomatic ones. The absence of the team’s own stool parasitological examination and of biomarkers of iron status and inflammation further limits the interpretation of the association between parasitosis and anemia and of the triple burden hypothesis. Specifically, neither stool microscopy nor molecular testing (e.g., PCR) was performed; parasite species and infection intensity were not characterized, helminth and protozoan infections could not be distinguished, and previous diagnoses could not be verified against medical records. These limitations may bias the magnitude, and potentially the direction, of the parasitosis–anemia association through exposure misclassification, and they preclude any species-specific inference. Similarly, anemia was defined exclusively on the basis of capillary hemoglobin measured with a point-of-care device; biomarkers that would allow the underlying cause of anemia to be identified—ferritin, transferrin saturation, soluble transferrin receptor and serum iron for iron deficiency; *C*-reactive protein and α1-acid glycoprotein for systemic inflammation; vitamin B12 and folate for megaloblastic anemia—were not measured. Consequently, the study cannot differentiate iron-deficiency anemia from anemia of inflammation, of parasitic origin, or of other causes, and the triple burden of malnutrition is offered only as a hypothesis to be tested in future studies in which both stool parasitological characterization and these biomarkers (with appropriate adjustment for inflammation) are available. We also acknowledge that the present design did not include a separately recruited reference group of lean/normal-weight children external to the community: rather, the WHO 2007 growth references served as the international standard against which weight status was classified, and the normal-weight subset of the sample itself (51/84, 60.7%) acted as the within-sample comparator group for the associations explored. Findings should therefore not be over-extrapolated and would benefit from confirmation in larger probabilistic samples with dedicated control groups. Fourth, adherence to the Mediterranean diet was approximated using a non-validated 12-item adaptation of the KIDMED index, so its results must be interpreted only as a descriptive indicator. Fifth, age was recorded in completed years, so that the z-scores were anchored to the midpoint of the year, and some perinatal variables showed data quality problems and were interpreted with particular caution. Finally, the performance of multiple comparisons and the restriction to a single locality and a single seasonal period advise considering the results as hypothesis-generating and limit their generalizability.

### 4.8. Future Directions

The results of this study invite confirmation and expansion through longitudinal designs and larger probabilistic samples that allow the limitations of power and representativeness to be overcome. The characterization of anemia and of the possible micronutrient deficiency would require the incorporation of stool parasitological confirmation and of biomarkers of iron status and of other micronutrients adjusted for inflammation, which would make it possible to directly test the triple burden hypothesis. Likewise, the use of validated dietary instruments and the evaluation of integrated double-duty interventions should combine the promotion of healthy eating and physical activity with water, sanitation, and hygiene measures, while deworming and supplementation should be guided by local parasitological surveillance and biomarker-based assessment.

## 5. Conclusions

This cross-sectional, exploratory study describes, through a multidimensional assessment, a mixed nutritional profile in a community sample of children and adolescents from a vulnerable peri-urban area of Limpio (Paraguay), compatible with a double burden of malnutrition. The predominance of excess weight and central adiposity coexisted with the persistence of stunting and with a high frequency of anemia, against a background of frequently reported intestinal parasitosis and low adherence to a high-quality dietary pattern. The most notable finding was the exploratory association between reported intestinal parasitosis and anemia, accompanied by a descriptive pattern involving absence of prior deworming; given the cross-sectional design and the lack of stool parasitological confirmation, this relationship must be interpreted as an association, and not as a causal sequence or as a risk factor in the strict sense.

The high burden of anemia, added to excess weight and growth deficits, suggests the possibility of an additional dimension of micronutrient deficiency that could bring the profile of this population closer to the emerging concept of a triple burden of malnutrition; this interpretation, however, is proposed as a hypothesis that would require confirmation through biomarkers of iron status and inflammation. Overall, the results support the appropriateness of addressing child and adolescent malnutrition from a comprehensive perspective that integrates the promotion of healthy eating and physical activity with the improvement of sanitation and access to safe water; decisions on deworming and on micronutrient supplementation should be guided by local parasitological surveillance and by biomarker-based assessment of micronutrient status, neither of which were available in the present study, in line with double-duty actions and using schools and community canteens as intervention platforms.

Owing to its exploratory nature, its non-probabilistic sampling, and its moderate sample size, these findings should be considered hypothesis-generating and do not allow representative inferences for the child and adolescent population of Limpio as a whole. Their confirmation will require longitudinal studies with larger and more representative samples and validated instruments, incorporating stool parasitological confirmation and the determination of biomarkers, and allowing the magnitude and directionality of the associations described here to be established. Even with these limitations, the study provides one of the first multidimensional characterizations of nutritional status in this community and offers a useful basis for guiding future public health interventions in vulnerable community contexts.

## Figures and Tables

**Table 1:** Community origin and assessment coverage of the participants evaluated in Limpio, Paraguay.

School Premises ^1^	Enrolled	Assessed	Coverage (%)
Premises 1	63	23	36.5
Premises 2	25	11	44.0
Premises 3	30	30	100
Premises 4	40	11	27.5
Premises 5	50	12	24.0
Premises 6	30	3	10.0
Total	238	90	37.8

^1^ The premises are presented in coded form to preserve institutional neutrality. As no random sampling was applied, the sample should be interpreted as an accessible community sample and not as a representative estimate of the child population of Limpio.

**Table 2:** Sociodemographic, perinatal, and environmental characteristics of the sample.

Variable	*n* (%) or Median [IQR]
Sex	
Boy	34 (37.8)
Girl	56 (62.2)
Age group (years)	
6–9	45 (50.0)
10–11	27 (30.0)
12–16	18 (20.0)
No. of siblings	2.0 [1–4]
Community	
Inmaculada Concepción	3 (3.3)
San Isidro	12 (13.3)
San Julián	23 (25.6)
Los Laureles	11 (12.2)
Comedor Mamá Virginia	30 (33.3)
Cacupemí	11 (12.2)
Type of housing	
Brick	74 (82.2)
Wood	9 (10.0)
Other	7 (7.8)
Water source	
Piped	68 (75.6)
Well	11 (12.2)
Bottled/communal/other	11 (12.2)
Exclusive breastfeeding ≥ 6 months	50 (55.6)
Physical exercise (yes)	66 (75.0)
Correct vaccination status	72 (84.7)
Reported intestinal parasitosis (yes)	21 (24.1)
Not dewormed	27 (30.7)
Micronutrient supplementation (yes)	2 (2.2)

IQR, interquartile range.

**Table 3:** Descriptives by sex (median [IQR]).

Variable	Boys (*n* = 34)	Girls (*n* = 56)	*p* *
Age (years)	9.0 [8.0–10.0]	10.0 [8.0–11.0]	0.167
Weight (kg)	33.9 [27.3–42.7]	37.6 [29.5–48.3]	0.242
Height (cm)	138.5 [129–149]	141.0 [133–150]	0.282
BMI (kg/m^2^)	17.6 [16.2–19.6]	18.6 [16.5–22.4]	0.233
BAZ (BMI-for-age z)	0.6 [−0.1–1.3]	0.7 [−0.1–1.4]	0.865
HAZ (height-for-age z)	0.2 [−1.1–0.8]	0.1 [−0.9–0.8]	0.917
Waist circumference (cm)	62.0 [57–73]	64.0 [60–72]	0.620
Waist-to-height ratio	0.46 [0.43–0.49]	0.46 [0.43–0.50]	0.720
Fat mass (%)	20.7 [16.0–29.0]	24.2 [18.7–31.0]	0.239
Hemoglobin (g/dL)	12.3 [11.2–12.9]	12.6 [11.9–13.3]	0.221
Glucose (mg/dL)	97.0 [89–101]	90.5 [86–97]	0.052
SaO_2_ (%)	98 [97–99]	98 [96–99]	0.593
SBP (mmHg)	109 [96–112]	110 [100–117]	0.165
DBP (mmHg)	60 [52–66]	62 [56–71]	0.242
HR (bpm)	91 [86–97]	93 [83–102]	0.546
Water intake (L/day)	2.0 [1.0–2.9]	2.0 [1.0–3.0]	0.563
Dietary quality (adapted KIDMED, 0–12)	2.0 [0–4]	3.0 [0–8]	0.140
Well-being (Ryff, 0–60)	49 [45–54]	48.5 [42–54]	0.891

* Mann–Whitney U test. IQR, interquartile range; BAZ, BMI-for-age z-score; HAZ, height-for-age z-score; SBP/DBP, systolic/diastolic blood pressure; HR, heart rate.

**Table 4:** Prevalence of the coexisting forms of malnutrition and associated conditions.

Condition	*n*/Total	Prevalence (%)	95% CI
Overweight or obesity (BAZ > +1 SD)	33/84	39.3	29.5–50.0
Obesity (BAZ > +2 SD)	14/84	16.7	10.2–26.1
Central obesity (WHtR > 0.5)	19/85	22.4	14.8–32.3
Anemia (WHO cut-offs)	20/74	27.0	18.2–38.1
Reported intestinal parasitosis	21/87	24.1	16.4–34.1
Stunting (HAZ < −2 SD)	7/85	8.2	4.0–16.0
Thinness (BAZ < −2 SD)	0/84	0.0	
Micronutrient supplementation	2/90	2.2	0.6–7.7

WHtR, waist-to-height ratio; BAZ, BMI-for-age z-score; HAZ, height-for-age z-score.

**Table 5:** Multivariable logistic regression model for anemia.

Variable	Unadjusted OR (95% CI)	*p*	Adjusted OR (95% CI)	*p*	Firth OR (95% CI)	*p*
Reported intestinal parasitosis (yes vs. no)	3.35 (1.09–10.28)	0.040	5.44 (1.44–20.51)	0.012	4.82 (1.33–17.49)	0.017
Female sex (vs. male)	0.49 (0.17–1.45)	0.195	0.26 (0.07–0.97)	0.045	0.29 (0.08–1.04)	0.058
Age (per year)	1.04 (0.80–1.35)	0.779	1.04 (0.78–1.39)	0.779	1.04 (0.78–1.38)	0.782

OR, odds ratio; CI, confidence interval. Dependent variable: anemia (yes/no). The unadjusted ORs come from bivariate models; the adjusted model and the Firth model include the three variables simultaneously. The analysis was based on 71 participants with complete data and 19 anemia events (6.3 events per variable). The Firth penalized logistic regression was applied as a sensitivity analysis, appropriate given the limited number of events per variable and the presence of cells with low frequencies.

## Data Availability

The raw data supporting the conclusions of this article will be made available by the authors on request.

## Supplementary Materials

The following supporting information can be downloaded at https://www.mdpi.com/article/10.3390/nu18132192/s1; Table S1: STROBE checklist for cross-sectional studies.

## Abbreviations

The following abbreviations are used in this manuscript:

BAZ	BMI-for-age z-score
BMI	Body mass index
CI	Confidence interval
DBP	Diastolic blood pressure
HAZ	Height-for-age z-score
HR	Heart rate
IOTF	International Obesity Task Force
IQR	Interquartile range
KIDMED	Mediterranean Diet Quality Index
OR	Odds ratio
SaO_2_	Arterial oxygen saturation
SBP	Systolic blood pressure
SD	Standard deviation
STROBE	Strengthening the reporting of observational studies in epidemiology
WHO	World Health Organization
WHtR	Waist-to-height ratio
