# Supplementary material for: Double Burden of Malnutrition and the Relationship Between Reported Intestinal Parasitosis and Anemia in School-Aged Children from a Peri-Urban Community of Limpio (Paraguay): A Cross-Sectional Study
Source: Nutrients. 2026 Jul 5;18(13):2192. doi: 10.3390/nu18132192 (PMC13363932; doi:10.3390/nu18132192)
Supplement: Supplementary file 1 [file nutrients-18-02192-s001.zip › nutrients-4372755-supplementary.pdf]

Double Burden of Malnutrition and the Association between Self-Reported Intestinal Parasitosis and Anemia among School-Aged Children in a Peri-Urban Community of Limpio, Paraguay: A Cross-Sectional Study

Supplementary Materials

Table S1. STROBE checklist for cross-sectional studies.

STROBE Statement—Checklist of items that should be included in reports of ***cross-sectional studies***

	Item No	Recommendation	Location in the manuscript
**Title and abstract**	1	(*a*) Indicate the study's design with a commonly used term in the title or the abstract	Title; Abstract ("cross-sectional study")
		(*b*) Provide in the abstract an informative and balanced summary of what was done and what was found	Structured abstract (Background/Objectives, Methods, Results, Conclusions)
**Introduction**			
Background/rationale	2	Explain the scientific background and rationale for the investigation being reported	Introduction (double burden, nutrition transition, anemia and parasitosis)
Objectives	3	State specific objectives, including any prespecified hypotheses	Introduction (final paragraph)
**Methods**			
Study design	4	Present key elements of study design early in the paper	Methods 2.1
Setting	5	Describe the setting, locations, and relevant dates, including periods of recruitment, exposure, follow-up, and data collection	Methods 2.1 (Limpio; 10–31 July 2024)
Participants	6	(*a*) Give the eligibility criteria, and the sources and methods of selection of participants	Methods 2.1 (ages 6–16; convenience sampling)
Variables	7	Clearly define all outcomes, exposures, predictors, potential confounders, and effect modifiers. Give diagnostic criteria, if applicable	Methods 2.3–2.7 (WHO 2007 criteria; WHO anemia thresholds; WHtR > 0.5)
Data sources/ measurement	8*	For each variable of interest, give sources of data and details of methods of assessment (measurement). Describe comparability of assessment methods if there is more than one group	Methods 2.2, 2.3, 2.5–2.7 (field booklet; devices; LMS method)
Bias	9	Describe any efforts to address potential sources of bias	Methods 2.2 and 2.8; Discussion 4.7
Study size	10	Explain how the study size was arrived at	Methods 2.1 (convenience sample; no formal sample-size calculation, exploratory study)

Quantitative variables	11	Explain how quantitative variables were handled in the analyses. If applicable, describe which groupings were chosen and why	Methods 2.7 and 2.9 (z-scores; age and sibship groups)
Statistical methods	12	(*a*) Describe all statistical methods, including those used to control for confounding	Methods 2.9 (Shapiro-Wilk; Mann-Whitney; Fisher; Spearman; logistic regression)
		(*b*) Describe any methods used to examine subgroups and interactions	Methods 2.9; Results 3.2 (comparisons by sex)
		(*c*) Explain how missing data were addressed	Methods 2.8 and 2.9 (no imputation; complete-case analysis per variable)
		(*d*) If applicable, describe analytical methods taking account of sampling strategy	Methods 2.1 and 2.9 (convenience sampling; no weighting; no population inference)
		(*e*) Describe any sensitivity analyses	Methods 2.9; Results 3.5; Table 5 (unadjusted, adjusted and Firth models)
**Results**			
Participants	13*	(a) Report numbers of individuals at each stage of study—eg numbers potentially eligible, examined for eligibility, confirmed eligible, included in the study, completing follow-up, and analysed	Methods 2.1 and Table 1 (238 accessible → 90 evaluated; 37.8%)
		(b) Give reasons for non-participation at each stage	Methods 2.1 (non-attendance, lack of authorization, incomplete measurements)
		(c) Consider use of a flow diagram	Coverage detailed by recruitment site in Table 1 (no flow diagram)
Descriptive data	14*	(a) Give characteristics of study participants (eg demographic, clinical, social) and information on exposures and potential confounders	Results 3.1–3.2; Tables 2–3
		(b) Indicate number of participants with missing data for each variable of interest	Tables 2–4 (n / denominators); Methods 2.8
Outcome data	15*	Report numbers of outcome events or summary measures	Results 3.4–3.5; Tables 4–5 (19 anemia events)
Main results	16	(*a*) Give unadjusted estimates and, if applicable, confounder-adjusted estimates and their precision (eg, 95% confidence interval). Make clear which confounders were adjusted for and why they were included	Results 3.5; Table 5
		(*b*) Report category boundaries when continuous variables were categorized	Methods 2.7; table and figure legends
		(*c*) If relevant, consider translating estimates of relative risk into absolute risk for a meaningful time period	Not applicable (prevalences with 95% CI reported in Table 4)

Other analyses	17	Report other analyses done—eg analyses of subgroups and interactions, and sensitivity analyses	Results 3.2, 3.5 and 3.6; Table 5
**Discussion**			
Key results	18	Summarise key results with reference to study objectives	Discussion (opening of Section 4)
Limitations	19	Discuss limitations of the study, taking into account sources of potential bias or imprecision. Discuss both direction and magnitude of any potential bias	Discussion 4.7
Interpretation	20	Give a cautious overall interpretation of results considering objectives, limitations, multiplicity of analyses, results from similar studies, and other relevant evidence	Discussion 4.1–4.6 (non-causal wording)
Generalisability	21	Discuss the generalisability (external validity) of the study results	Discussion 4.7 (convenience sample; single locality and season)
**Other information**			
Funding	22	Give the source of funding and the role of the funders for the present study and, if applicable, for the original study on which the present article is based	“Funding” section (manuscript back matter)

*Give information separately for exposed and unexposed groups.

Note: An Explanation and Elaboration article discusses each checklist item and gives methodological background and published examples of transparent reporting. The STROBE checklist is best used in conjunction with this article (freely available on the Web sites of PLoS Medicine at http://www.plosmedicine.org/, Annals of Internal Medicine at http://www.annals.org/, and Epidemiology at http://www.epidem.com/). Information on the STROBE Initiative is available at www.strobe-statement.org.
